# Supplementary material for: Flavonoids, Phenolic Acids, and Tannin Quantities and Their Antioxidant Activity in Fermented Fireweed Leaves Grown in Different Systems
Source: Plants (Basel). 2024 Jul 12;13(14):1922. doi: 10.3390/plants13141922 (PMC11281143; doi:10.3390/plants13141922)
Supplement: Supplementary file 1 [file plants-13-01922-s001.zip › plants-3079946-supplementary.pdf]

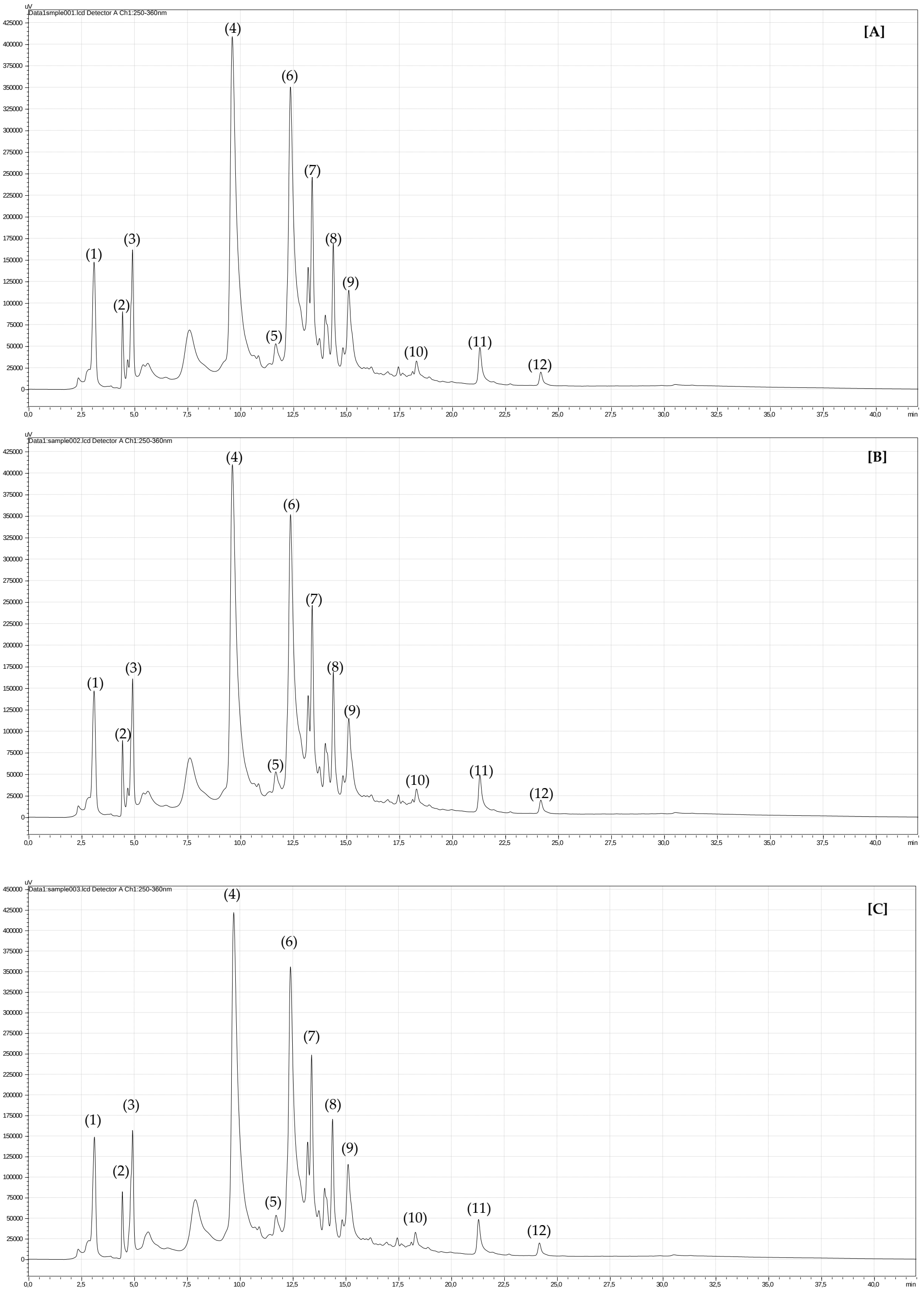

Figure S1. Chromatograms of fireweed produced by natural way [A] Control, [B] fermented 24h, [C] fermented 48h

(1) gallic acid, (2) oenothein B, (3) chlorogenic acid, (4) p-coumaric acid, (5) benzoic acid, (6) quercetin-3-O-rutinoside, (7) ellagic acid, (8) myricetin, (9) quercetin, (10) luteolin, (11) kaempferol, (12) quercetin-3-O-glucoside

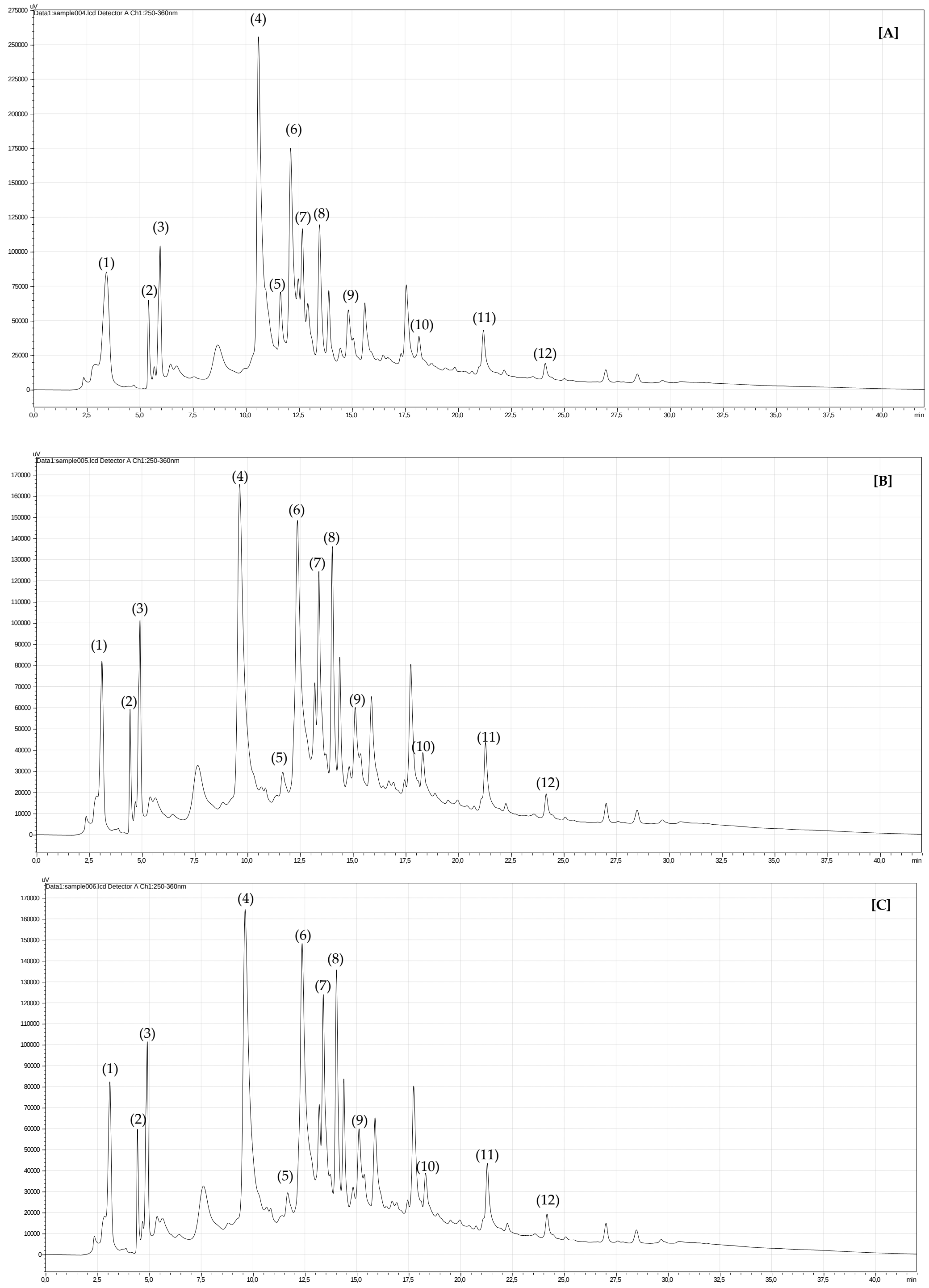

Figure S2. Chromatograms of fireweed produced by organic way [A] Control, [B] fermented 24h, [C] fermented 48h

(1) gallic acid, (2) oenothein B, (3) chlorogenic acid, (4) p-coumaric acid, (5) benzoic acid, (6) quercetin-3-O-rutinoside, (7) ellagic acid, (8) myricetin, (9) quercetin, (10) luteolin, (11) kaempferol, (12) quercetin-3-O-glucoside

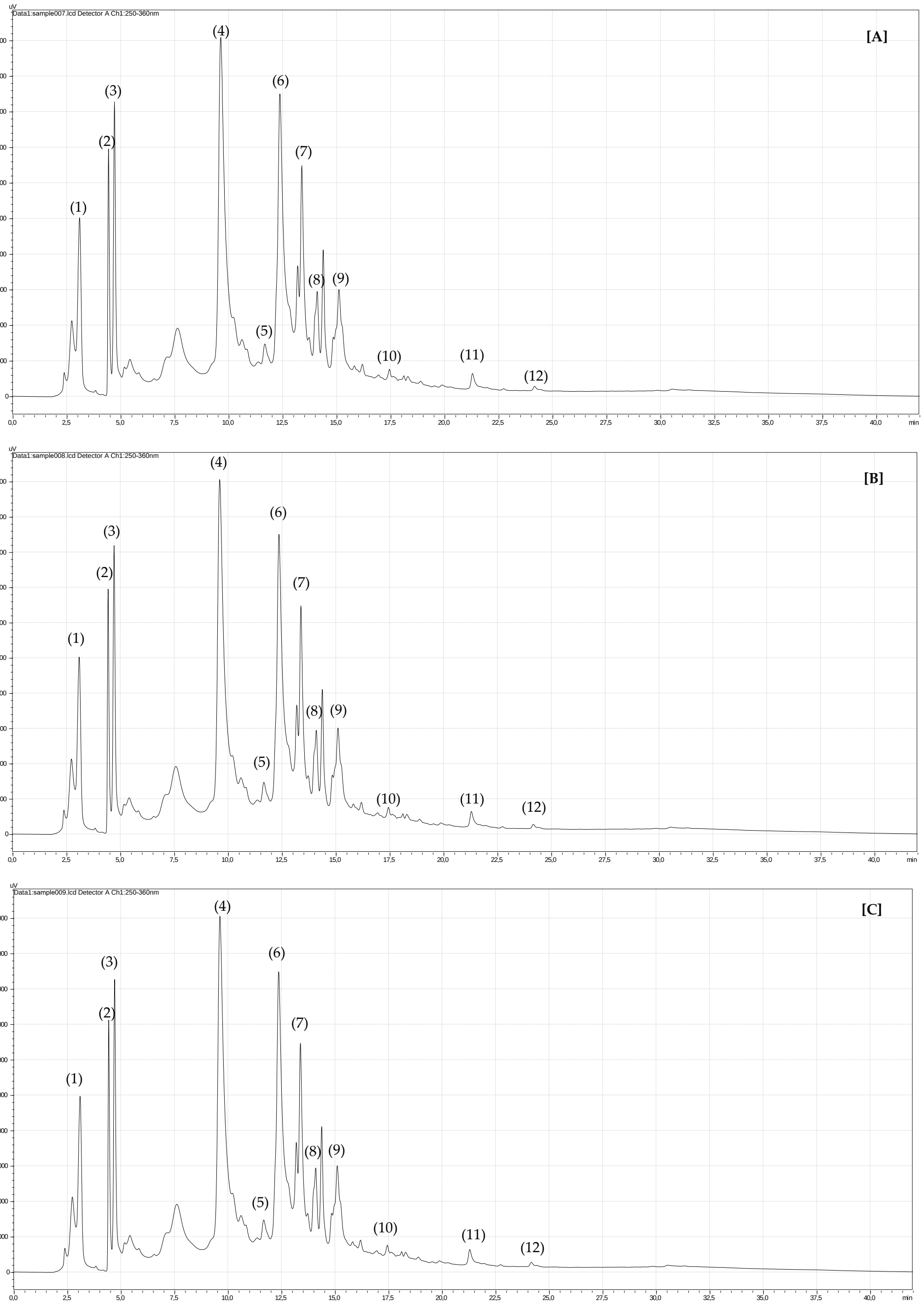

Figure S3. Chromatograms of fireweed produced by biodynamic way [A] Control, [B] fermented 24h, [C] fermented 48h

(1) gallic acid, (2) oenothein B, (3) chlorogenic acid, (4) p-coumaric acid, (5) benzoic acid, (6) quercetin-3-O-rutinoside, (7) ellagic acid, (8) myricetin, (9) quercetin, (10) luteolin, (11) kaempferol, (12) quercetin-3-O-glucoside
